# CLINICAL AND RADIOGRAPHIC EVALUATION OF ELBOWS FROM SPINAL CORD INJURIED PATIENTS

**DOI:** 10.1590/1413-785220162402154665

**Published:** 2016

**Authors:** Fabiana de Godoy Casimiro, Gabriel Faria de Oliveira, Pedro Henrique de Magalhães Tenório, Isabella da Costa Gagliardi, Américo Zoppi, Alberto Cliquet

**Affiliations:** 1. Universidade Estadual de Campinas, Faculdade de Ciências Médicas, Department of Orthopedics and Traumatology, Campinas, SP, Brazil

**Keywords:** Elbow, Spinal cord injuries, Osteoarthritis, Pain

## Abstract

**Objectives:**

: To evaluate clinically and radiologically the elbows of spinal cord injured patients and compare them to the control group.

**Methods:**

: Twenty patients (10 paraplegics and 10 tetraplegics) were clinically evaluated through assessment of pain scale, measurement of active and passive range of motion, degree of muscle strength and MEPS score. They were also submitted to bilateral plain radiography of the elbows. Both groups were compared to the control group.

**Results:**

: Four paraplegic and three tetraplegic patients referred mild to moderate, sporadic and motion related pain. The control group was asymptomatic. No statistic significant difference was found in passive range of motion among the three groups. The tetraplegic group showed a lower active range of motion as well as lower MEPS score as compared to the control group. Equal number of patients in the spinal cord injured patients had radiological abnormalities, but those were more severe in the tetraplegic group.

**Conclusion:**

: Spinal cord injured patients presented clinical and radiological elbow abnormalities, which were more evident on tetraplegics. Level of Evidence III, Case Control.

## INTRODUCTION

Spinal cord injuries have been increasing over time, having as primary cause accidents with motor vehicles, followed by injuries due to firearms and falls from heights.^1^ They are more frequent in men aged between 15-40 years old.^2^ The increase in the number of patients with secondary sequelae to spinal cord injuries is due to a highly effective pre-hospital care, which involves control of airways and bleeding, and also spine stabilization.^3^ These precautions allow patients involved in high-energy trauma to survive. Therefore, people who formerly would evolve to death, nowadays survive, but often acquire irreversible consequences, among them spinal cord injuries.

Studies show that the average incidence of traumatic spinal cord injury ranges from 15 to 40 cases per million inhabitants, but in some regions it can reach up to 246 cases/million inhabitants.^4^ In Brazil, the annual incidence of traumatic spinal cord injuries is around 40 cases/million inhabitants, which represents about 6-8 thousand new cases per year.^1^

Tetraplegic and paraplegic patients start to develop physical adaptations in order to perform everyday activities, such as locomotion and transfer. As a consequence, the upper limbs are, therefore, required more intensively, leading to the occurrence of clinical and anatomical changes.

Some studies suggest that regarding upper limbs, shoulder pain is the most prevalent among patients with spinal cord injury, followed by elbow, wrist and hand pain.^5^^,^ 6

The aim of this study was to evaluate clinically and radiographically elbows of spinal cord injured patients (quadriplegics and paraplegics) and compare the findings to healthy subjects (control group), confronting the data found in the literature.

## MATERIALS AND METHODS

Twenty patients being treated at the Laboratory of Biomechanics and Rehabilitation of the Locomotor System of *Hospital das Clínicas da Faculdade de Ciências Médicas da Universidade Estadual de Campinas*, Unicamp, SP, Brazil were divided into two groups: 10 paraplegics and 10 quadriplegics. The 20 individuals used solely wheelchairs for locomotion. These patients were evaluated and compared with 10 healthy subjects in the control group. The study project was analyzed by the Ethics Committee of the institution and accepted under CCAE number 23257613.4.0000.5404

The evaluation consisted of a questionnaire with open questions on complaints to the elbows, where patients could manifest themselves freely. In cases where there were reports of pain, patients were asked to classify it as mild, moderate or severe, continuous or sporadic and handedness.

Subsequently, the measurement of range of motion (ROM) for flexion-extension and active and passive pronosupination bilaterally, was performed using a goniometer. Muscle strength was classified according to criteria of the American Spinal Injury Association (ASIA), 7 ranging from zero (no muscle contraction) to 5 (normal motricity and strength). The MEPS questionnaire (Mayo Elbow Performance Score) was also applied.^8^

Finally, all patients underwent plain radiographs of the elbow in the anteroposterior and lateral (profile) views, in the same RX device and all images were observed by the same computerized radiographic method excluding any possible biases. The images did not show identification on the group they belonged (tetraplegic, paraplegic or control groups) and were evaluated by two members of the Shoulder and Elbow Surgery Society, independently, all at once. Divergent assessments were reassessed by the two experts together in order to reach a consistent classification. The images were classified from zero (no change) to four (severe degenerative changes), according to description provided by Morrey et al.^9^

All patients were instructed on the phases of the study. All participants received and signed the Free and Informed Consent Term, which was also approved by the Unicamp Ethics Committee.

## RESULTS

Thirty subjects were divided into groups - paraplegics, quadriplegics and control group (CG) - with 10 individuals each, eight men and two women. The mean age was 45.4 years old in the paraplegic group, 39.9 years old in the quadriplegic group of and 32.5 years old in the control group. (Table 1) The mean time of injury was 15.7 years in the paraplegic group and 14.1 years in the quadriplegic group. (Table 1) Among the paraplegics we observed two individuals with mild pain and two with moderate pain. Among quadriplegics, two reported mild pain and one reported moderate pain. All pain complaints were sporadic and movement related. There were no complaints of severe pain among all individuals and no pain whatsoever in the control group. Patients who reported pain were evaluated clinically and instructed by the shoulder and elbow orthopedic specialist on how to manage pain. All patients had some degree of pain improvement after the instructions.

**Table 1.** Quantification of time of injury and age of individuals, in years.

	**Tetraplegic**	**Paraplegic**	**Control group**
Mean time of injury (Min - Max)	14.1 (2-27)	15.7 (9-28)	0
Mean age (Min - Max)	39.9 (31-48)	45.5 (28-68)	32.5 (27-50)

As for active ROM, we noticed that the quadriplegic patients tend to have lower ROM as compared to paraplegics and the control group, as shown in Table 2. Assessment of passive ROM showed no significant change between the three groups. (Table 3)

**Table 2.** Results of active ROM.

	**Tetraplegic**	**Paraplegic**	**Control group**
Active flexion	Right 127.5°	Right 147°	Right 148°
	Left 142°	Left 146.5°	Left 148°
Active Pronation	Right 53°	Right 73°	Right 73°
	Left 48.5°	Left 72.5°	Left 73°
Active Supination	Right 55.5°	Right 83°	Right 82°
	Left 59°	Left 82.5°	Left 82.5°

**Table 3.** Results of passive ROM.

	**Tetraplegic**	**Paraplegic**	**Control group**
Passive flexion	Right 145.5°	Right 148°	Right 148°
	Left 145.5°	Left 148°	Left 149.5°
Passive pronation	Right 73°	Right 74°	Right 74.5°
	Left 73.5°	Left 73.5°	Left 74.5°
Passive supination	Right 79°	Right 84°	Right 84°
	Left 78.5°	Left 83.5°	Left 84°

Regarding the evaluation of muscle strength, we found that paraplegic patients show the same muscle strength than the control group, and that quadriplegics have a decreased degree of bilateral strength. (Table 4)

**Table 4.** Assessment of muscle strength.

	**Tetraplegic**	**Paraplegic**	**Control group**
Right Biceps	4.2	5	5
Left Biceps	4.1	5	5
Right Triceps	3.3	5	5
Left Triceps	3.6	5	5

In the radiographic evaluation of the elbows, no subject from the control group exhibited abnormalities in the images. Some paraplegic and quadriplegic patients had radiographic changes, the left side being slightly more affected than the right side. The tetraplegics were more severely affected according to the rating scale from Morrey et al.^9^ The results are shown in Tables 5 and 6.

**Table 5.** Quantity of individuals with radiographic alterations. Right elbow.

**Alteration**	**Tetraplegic**	**Paraplegic**	**Control group**
Grade 0	6	6	10
Grade 1	0	2	0
Grade 2	2	2	0
Grade 3	2	0	0
Grade 4	0	0	0

**Table 6.** Quantity of individuals with radiographic alterations. Left elbow.

**Alteration**	**Tetraplegic**	**Paraplegic**	**Control group**
Grade 0	5	5	10
Grade 1	1	2	0
Grade 2	3	3	0
Grade 3	1	0	0
Grade 4	0	0	0

The MEPS average score was 100 on CG, 99.5 (95-100) for paraplegics and 81.0 (40-100) in quadriplegics. 

## DISCUSSION

Patients with spinal cord injury have physical limitations that often prevent them from having a normal social interaction, making it difficult interpersonal relationships with family, friends and at work.^10^

The elbow is a ginglymus joint, intolerant to trauma, with high propensity to stiffness and degeneration. Formed by the distal humerus with the trochlea articulating with the olecranon and the capitulum articulating with the radial head. It is surrounded by soft tissue such as anterior and posterior capsule, medial and lateral ligament complex and flexor extensor, pronator and supinator muscles.^11^

Morrey et al.^12^ reported that a functional range of motion during daily activities is between 30° and 130° for flexion-extension and 100° for pronosupination (50° in each direction). The normal range of motion of elbow flexion is 140° ± 5°, pronation is 75°, and mean supination is 80°. Full normal extension should be 0°.^13^

In normal individuals, the upper limbs are used primarily for prehension. In spinal cord injured individuals the joints are turned into load-bearing joints, as they are required intensely during transfer, propelling the wheelchair and use of crutches, when possible.^14^ Moreover, due to the need of the sitting position, many daily activities need to be performed with the arm above the head, resulting in muscle imbalance and overload.^15^ This overload affects one joint not designed to withstand such loads and possibly presents a muscle imbalance depending on the level of injury, resulting in pain and further, some degree of joint destruction. 

The primary osteoarthritis of the elbow mainly affects middle-aged men with higher functional demand on upper limbs. Clinically they may present with pain, muscle weakness and stiffness. X-rays show bone osteophytes mainly in olecranon and coronoid, decreased joint space and, finally, articular degeneration.^16^^,^ 17

**Figure 1.**
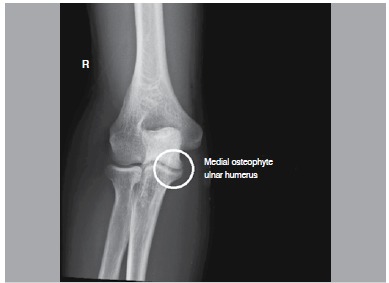
Radiographic alteration grade 1. Presence of medial osteophyte shown in the anteroposterior X-ray.

**Figure 2.**
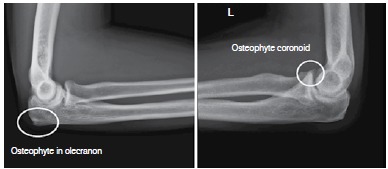
Radiographic alteration grade 2. Presence of osteophyte in olecranon and coronoid, shown in profile X-ray.

**Figure 3.**
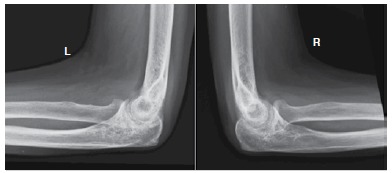
Radiographic alteration grade 3. Alterations on the articular surface can be seen, but the shape of the radial head is maintained and there are no loose bodies.

**Figure 4.**
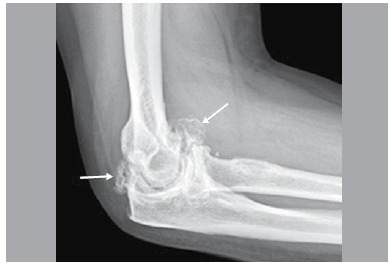
Radiographic alterations grade 4. Articular destruction and loose bodies (arrows).

There is no report in the literature of a classification for elbow osteoarthritis, however, Morrey et al.^9^ refers to the order of appearance of radiographic changes in such cases. First comes a medial osteophytes (humerus-ulnar) displayed on anteroposterior X-ray, classified as grade 1 (Figure 1) Then the osteophytes in the olecranon and coronoid arise, visualized in profile radiographs, classified as grade 2. (Figure 2) Later, changes resulting from joint cartilage wear can be seen, classified as grade 3. (Figure 3) Finally, loose bodies and deformity in the radial head are seen (Figure 4). We use this radiographic description of the changes, as described by Morrey, to classify the radiographic findings of patients in the present study.

The MEPS score evaluates, indirectly, the quality of life of individuals, based on the quantification of four criteria: pain, range of motion, joint stability and daily life activities.^8^ The final sum allows to assess the elbow performance. In our study we observed that paraplegic patients showed an excellent score, as well as the control group (> 90), while quadriplegic patients had a mean of 81, considered as good. This lower figure was mainly due to the limitations t perform daily activities. No patient presented joint instability.

Using the Spearman correlation coefficient, we concluded that the greatest degree of muscle strength of the biceps and triceps is directly related to a better MEPS score (coefficient 0.75, *p*<0.0001). There was also a significant correlation between

active flexion and MEPS (0.608, *p*=0.0004) and active supination and MEPS (0.597, *p* = 0.0005). Active pronation and passive ROM have little correlation with MEPS score. The Spearman correlation coefficient ranges from -1, when the correlation between the variables compared is negative, to +1 when the correlation is positive, total. The null value (zero) means no correlation.^18^

## CONCLUSION

This study demonstrated that spinal cord injured patients have clinical and radiographic changes in the elbows, probably due to the overload applied to this joint. Despite having discrete pain complaints, we know that muscle imbalance with diminishing strength can lead to joint instability and consequent degeneration. The fact that there are few studies published on elbows of injured patients leads us to rethink the importance of giving more attention to these patients, especially regarding their quality of life. Therefore, we need to further study the ills that afflict them and try, when possible, to provide greater physical well-being by preventing or *delaying* the onset of the changes secondary to spinal cord injury.
